# Acetazolamide Mitigates Intracranial Pressure Spikes Without Affecting Functional Outcome After Experimental Hemorrhagic Stroke

**DOI:** 10.1007/s12975-018-0663-6

**Published:** 2018-09-17

**Authors:** Michael R. Williamson, Cassandra M. Wilkinson, Kristen Dietrich, Frederick Colbourne

**Affiliations:** 1 Neuroscience and Mental Health Institute, University of Alberta, Edmonton, Canada; 2 P217 Biological Sciences Building, Department of Psychology, University of Alberta, Edmonton, AB T6G 2E9 Canada

**Keywords:** Acetazolamide, Compliance, Intracerebral hemorrhage, Intracranial pressure, Stroke

## Abstract

Increased intracranial pressure (ICP) after stroke can lead to poor outcome and death. Novel treatments to combat ICP rises are needed. The carbonic anhydrase inhibitor acetazolamide diminishes cerebrospinal fluid (CSF) production, reduces ICP in healthy animals, and is beneficial for idiopathic intracranial hypertension patients. We tested whether acetazolamide mitigates ICP elevations by presumably decreasing CSF volume after collagenase-induced striatal hemorrhage in rats. We confirmed that acetazolamide did not adversely affect hematoma formation in this model or physiological variables, such as temperature. Then, we assessed the effects of acetazolamide on ICP. Lastly, we tested the effects of acetazolamide on behavioral and histological outcome. Acetazolamide reduced the magnitude and occurrence of short-timescale ICP spikes, assessed as disproportionate increases in ICP (sudden ICP increases > 10 mmHg), 1-min peak ICP, and the magnitude of spikes > 20 mmHg. However, mean ICP was unaffected. In addition, acetazolamide reduced ICP variability, reflecting improved intracranial compliance. Compliance measures were strongly correlated with high peak and mean ICP, whereas ipsilateral hemisphere water content was not correlated with ICP. Despite effects on ICP, acetazolamide did not improve behavioral function or affect lesion size. In summary, we show that intracerebral hemorrhage creates an impaired compliance state within the cranial space that can result in large, transient ICP spikes. Acetazolamide ameliorates intracranial compliance and mitigates ICP spikes, but does not improve functional outcome, at least for moderate-severity ICH in rats.

## Introduction

Intracranial pressure (ICP) elevations after stroke contribute to mortality and poor outcome [1]. The magnitude and duration of ICP elevations, frequency of ICP spikes, and intactness of intracranial compliance are predictors of outcome [1–4]. Existing treatments are highly invasive (e.g., shunt or craniectomy) or mainly target cerebral edema (e.g., mannitol) [5]. However, while edema is an important contributor to raised ICP, it is not the only cause. For instance, preclinical data has shown that edema can be significantly reduced without affecting ICP [6], and ICP can be lowered without a change in brain water content [7]. Perhaps due to their rather narrow focus, current treatments are not consistently effective [8, 9]. Thus, there is a need for novel, minimally invasive treatments to reduce post-stroke ICP.

Space-occupying lesions, such as intracerebral hemorrhages (ICH), can increase ICP by adding to the volume of the intracranial space [10]. Reducing intracranial volume may be an effective strategy to counter high ICP. One possible method to safely reduce the total volume of intracranial contents is by diminishing cerebrospinal fluid (CSF) volume. Here, we tested whether the FDA-approved carbonic anhydrase inhibitor acetazolamide, well-known to decrease CSF production by altering bicarbonate transport in choroid plexus [11, 12], would attenuate ICP elevations after collagenase-induced ICH. This ICH model significantly raises ICP for at least several days [7, 13], whereas the other common ICH model, whole blood infusion, only modestly (30-min peak ICP < 15 mmHg) and transiently (~ 24 h) raises ICP [14].

In order to continually measure ICP while avoiding possible artifacts from restraint or anesthesia, we used head-implanted telemetric pressure probes connected to the epidural space. We have repeatedly used this technique to monitor minute-by-minute ICP changes in freely moving rats over several days [7, 13–16]. In addition, we tested whether acetazolamide affects hematoma formation because it can acutely increase cerebral blood flow [17], and early interventions can aggravate bleeding after collagenase-induced ICH [18]. Finally, we assessed effects of acetazolamide on behavioral and histological outcome. We evaluated behavioral function with two tasks sensitive to impairments in this ICH model [19]. The Neurologic Deficit Scale was used to assess outcome as early as 3 days after ICH (when ICP is raised) and as late as 28 days. The staircase reaching task was used to probe long-term function from 23 to 27 days post-ICH.

## Materials and Methods

This study conforms to the RIGOR guidelines for effective translational research [20].

### Subjects

Seventy male Sprague-Dawley rats (typically ~ 250–350 g, ~ 8–12 weeks old; ~ 500–600 g for experiments measuring blood pressure and temperature) from the University of Alberta biosciences colony or Charles River Laboratories were used. Source of animals was consistent within experiments. Note that the local colony is bred from rats obtained from Charles River. Animals were housed (individually, except for the behavioral experiment where they were grouped four per cage) in temperature and light-controlled rooms (lights on 7 a.m.–7 p.m.) with free access to water. Food access was unrestricted except during training and testing on the staircase task when animals were restricted to 90% free-feeding weight to encourage reaching. Animals were randomly assigned to groups after surgery using a random number generator. Testing and data analysis were done blinded. Sample sizes were based on previous work and a priori power analyses. Eight animals per group were calculated to give 80% power to detect a 35% change in hematoma volume (SD = 10 μL). Nine animals per group were calculated to give 85% power to detect a 30% reduction in peak ICP (SD = 5 mmHg) [7, 13]. Twelve animals per group were calculated to give 85% power to detect a 25% relative difference in reaching success on the staircase task (SD = 15%).

### Experimental Design

First, we assessed the safety profile of acetazolamide after ICH. In four animals, we monitored core temperature via telemetry. In three animals, we monitored blood pressure via telemetry. In four anesthetized animals, we acutely monitored blood pressure, heart rate, oxygen saturation, and blood glucose. In another cohort, hematoma volume was measured 24 h post-ICH (*N* = 8 per group, drug vs. vehicle). Next, we measured ICP continually via telemetry after ICH until brain water content was determined at 72 h (*N* = 10 acetazolamide-treated, 9 vehicle-treated). In the final experiment, animals were first trained on behavioral tests (*N* = 12 per group). Then, the effects of acetazolamide on behavioral and histological outcome were assessed up to 28 days post-ICH.

### Drug Dosage

Acetazolamide (Sigma, Oakville, ON; 50 mg/kg (~ 0.5 mL total volume) IP in 15% DMSO in saline, pH adjusted to 9 with NaOH to increase solubility [21], 2 mL/kg) or vehicle was given beginning 3 h after ICH. This dose of acetazolamide reduces CSF production to ~ 55% in Sprague-Dawley rats [11, 12] and is similarly effective in other species [11, 22]. This appears to be the maximal effect due to the presence of a subset of acetazolamide-insensitive carbonic anhydrases [23]. Therefore, higher doses would not confer greater inhibition of CSF production. This dose is comparable to the typical daily dose in humans based on allometric scaling [24, 25]. We delayed administration to 3 h post-ICH to match clinically feasible delivery and avoid administration during the majority of active bleeding, which occurs largely during the first hour after collagenase infusion [26]. In experiments with survival times > 24 h, drug or vehicle was administered 3 h after ICH and three more times at 24-h intervals. Dose intervals of 24 h were chosen based on a previous study that showed reduced ventricle volume for at least 24 h after a single 30 mg/kg IP dose [27]. Based on previous observations, ICP appears to return to the normal range by the fourth to fifth day after collagenase-induced ICH [7]. Therefore, acetazolamide would be active throughout the period of raised ICP with this dosing regimen. Naïve rats maintained normal body weight throughout and after this dosing regimen (data not shown).

### Intracerebral Hemorrhage

**Fig. 1:**
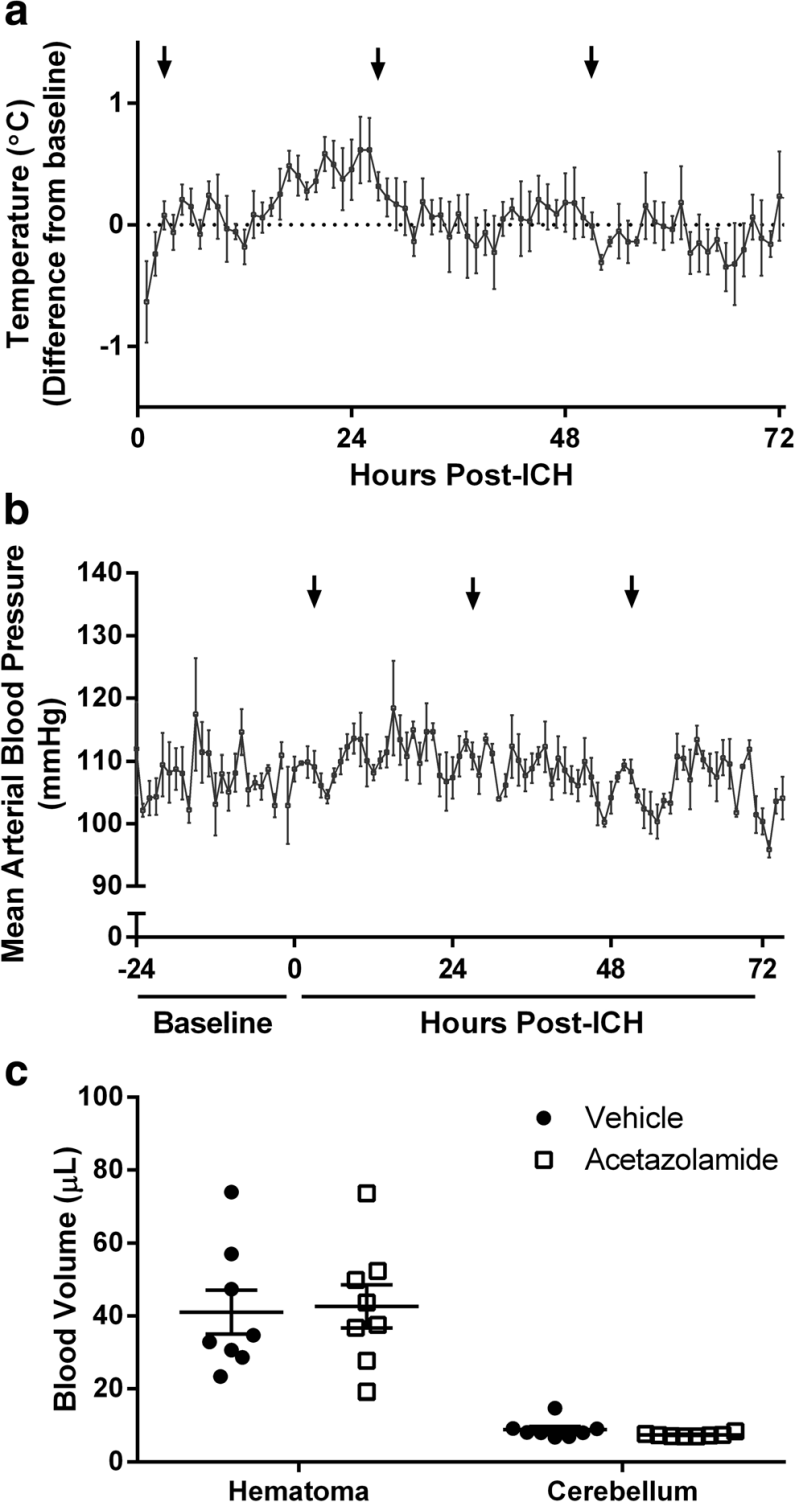
Acetazolamide (50 mg/kg, IP) does not affect core temperature (**a**, *n* = 4) or blood pressure (**b**, *n* = 3) after ICH. Both parameters were measured by implanted telemetry probes. Arrows indicate time of drug administration. **c** Acetazolamide given 3 h post-ICH did not affect hematoma volume measured at 24 h

Isoflurane (4% induction, ~ 2% maintenance in 70% N_2_O, balance O_2_) was used for anesthesia. Hemorrhage was induced by striatal infusion of bacterial collagenase (Type IV-S, Sigma; 0.2 U, 0.2 U/μL in saline; 0.5 mm anterior, 3.5 mm lateral from Bregma, 6.5 mm below skull surface) [28]. Rectal temperature was maintained at 37 °C with a heated pad during surgery, but not afterwards as ICH rats tend to regulate their temperature well (Fig. 1a). Animals were given soft, wet peanut butter mash mixed with rat chow after surgery to minimize food and water intake deficits [29]. Collagenase was infused into the right striatum in the first two experiments and contralateral to the preferred limb for reaching in the third experiment.

### Hematoma Volume Assay

Hemispheric blood volume was determined with a spectrophotometric hemoglobin assay as before [18, 28]. Hematoma size was calculated as ipsilateral-contralateral blood volume. Cerebellar blood volume served as a control.

### Measurement of Physiological Parameters

Calibrated TA10TA-F40 probes (Data Sciences Int., St. Paul, MN) were inserted into the peritoneal space to monitor temperature as described [14]. Data was collected with DataQuest ART software (v2.3, Data Sciences Int.). Arterial blood gases were assessed at the time of euthanasia in these animals (ABL800 flex, Radiometer).

Calibrated PA-C10 probes were inserted into the femoral artery to monitor blood pressure as described [13].

Oxygen saturation, glucose, heart rate, and blood pressure were measured in anesthetized rats. Pulse oximetry was measured using with a MouseOx Plus sensor (Starr Life Sciences) on the hind paw. The tail artery was cannulated and connected to a pressure transducer (BP-1, World Precision Instruments). Blood glucose was measured from tail capillary blood samples (Contour Next One).

### Intracranial Pressure Measurement

ICP and animal movement were measured with PA-C10 telemetric pressure probes (Data Sciences Int.) implanted in the epidural space immediately after collagenase infusion [7, 13–16]. Probes were calibrated by Data Sciences Int. prior to surgery and measurements adjusted as described [7, 14, 16]. Data were collected every minute with DataQuest ART software. ICP data were exported from ART and then processed with Excel (Microsoft) and MATLAB (R2016a, MathWorks). Data from all animals was processed simultaneously in a blinded manner. Movement and electrical interference artifacts were removed prior to analysis [14]. Disproportionate increases in ICP (DIICP) were evaluated based on previous work [3, 30]. DIICP events were defined as sudden ICP increases > 10 mmHg lasting at least 3 min. The baseline reference was defined as the average ICP over the previous 60 min. Other analyses included mean ICP over 12-h epochs, peak 1-min and 30-min ICP, average magnitude of spikes above 20 mmHg (the clinical definition of intracranial hypertension [5] and a threshold over which the risk of death in rats increases [13, 15]), and ICP variability (magnitude of ICP change between 1- and 30-min timeframes). High ICP variability reflects poor intracranial compliance [2, 4]. Analysis of standard deviations yielded similar results (not shown), but such a method of analysis does not convey individual change events and thus may tend to mask brief or uncommon but important events [3].

### Brain Water Content Determination

Water content was determined with the wet-dry method in tissue blocks that encompassed 2 mm anterior to 4 mm posterior from the infusion site [14]. Tissue weights were recorded before and after baking at 100 °C for 24 h. Water content was calculated as a percentage of the wet tissue weight. Cerebellum served as a control.

### Behavioral Assessment

Animals were trained and tested on the Montoya staircase and Neurologic Deficit Scale (NDS) tasks as described previously [14, 19]. The NDS task is comprised of subtests examining forepaw flexion, hindlimb retraction, spontaneous circling, beam walking, and forepaw grasp. Scores from each subtest were combined to form composite scores, which range from 0 (no deficits) to 14 (largest deficits). Baseline NDS testing was done in a single session after sufficient subtest training. Post-ICH NDS testing was done on days 3, 7, 14, 21, and 28. A single day of staircase training or testing included two sessions separated by at least 4 h. Baseline function on the staircase task was defined as the average success rate with the preferred paw over the final 3 days of training. Reaching data from animals that failed to maintain an average success rate of 9 out of 21 pellets per session over the final 3 training days were excluded. Reaching ability was probed on days 23 to 27 after ICH.

### Histology

Animals were overdosed with sodium pentobarbital (~ 100 mg/kg IP) and perfused with 0.9% saline followed by 10% neutral-buffered formalin. Brains were post-fixed and then cryoprotected in 30% sucrose prior to sectioning with a cryostat. Forty-micrometer sections spaced 200 μm apart and extending through and beyond the lesion were stained with cresyl violet. Lesion volume was assessed as described previously [19]. In short, lesion volume = volume of intact hemisphere − volume of injured hemisphere. This analysis accounts for necrosis, cavity formation, and ventriculomegaly.

### Statistical Analysis

Data were analyzed with GraphPad Prism (v. 6.0, GraphPad Software Inc., La Jolla, CA). Independent means were compared with unpaired Student’s *t* tests. Variance was compared with *F*-tests, and corrections (i.e., Welch’s correction) were applied when appropriate. Normality was assessed with D’Agostino and Pearson omnibus tests. Mean ICP over time and staircase data were analyzed with repeated measures ANOVA. Cumulative probabilities of ICP change events were compared with Kolmogorov-Smirnov tests. Linear regressions assessed the relationships between variables. Ordinal NDS data were assessed with Mann-Whitney *U* tests. One-tailed tests were used when comparing univariate effects of acetazolamide versus vehicle on ICP because all past work has shown that acetazolamide consistently reduces CSF production and ICP, and there is no obvious mechanism for acetazolamide to increase ICP. All other tests were two-tailed. Data are presented as mean ± SEM. Statistical significance was set at *P* < 0.05.

## Results

There was no mortality. One animal per group was excluded from ICP and movement analysis in the second experiment due to probe battery failure. Brain water content measurements from those animals were included. Staircase data from three vehicle-treated animals were excluded from the third experiment due to failure to meet minimum success criteria during training. NDS and lesion volume data from those animals were included.

**Fig. 2:**
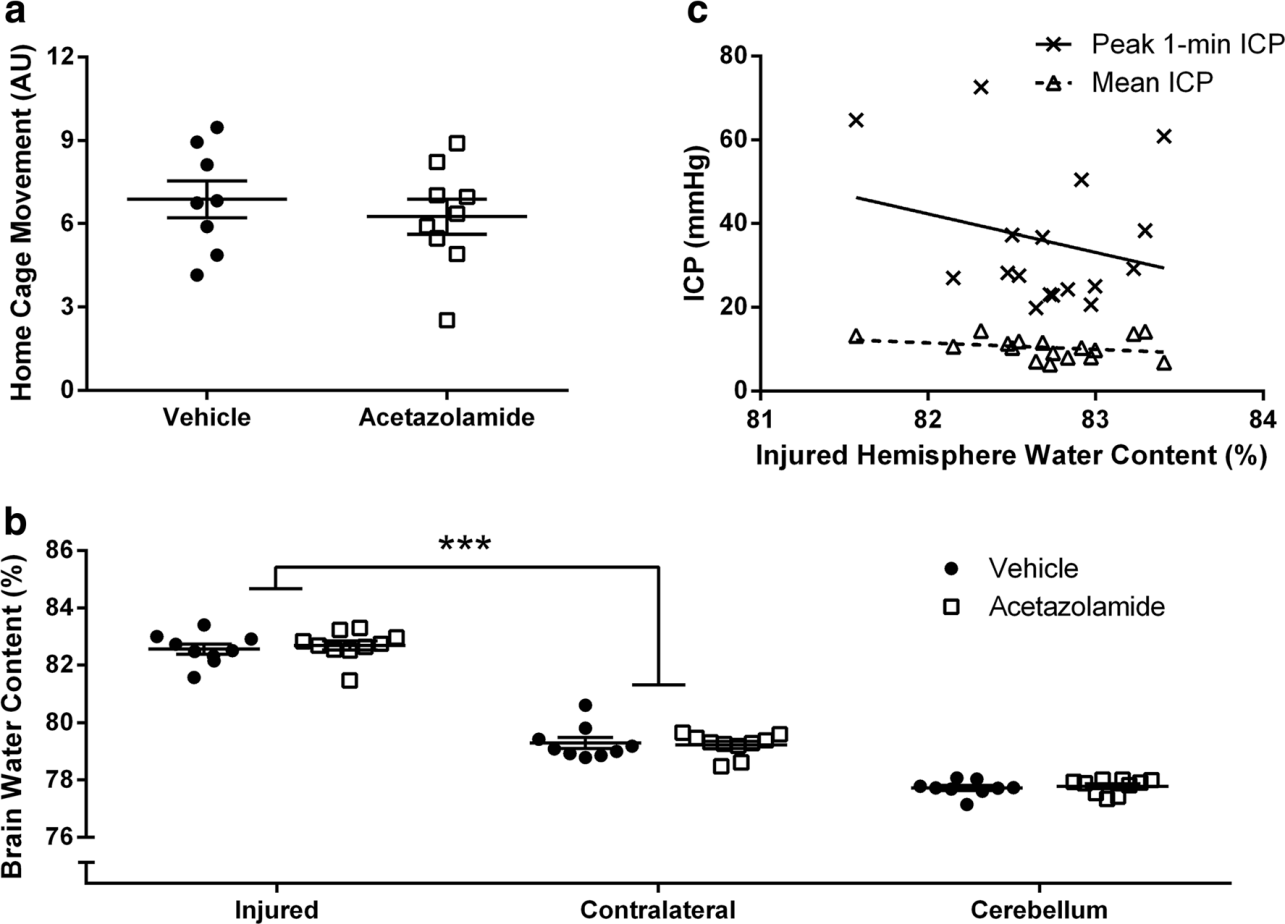
Home cage activity and edema after ICH. **a** Acetazolamide did not affect home cage movement. **b** ICH significantly increased brain water content within in the injured hemisphere, but there was no effect of acetazolamide. Measurements were made on 6-mm tissue blocks encompassing the hemorrhage in the injured hemisphere, an equivalent area in the contralateral hemisphere, and the entire cerebellum (see “Materials and Methods”). **c** Brain water content in the injured hemisphere was not correlated with mean or peak ICP (*r* = − 0.347 to − 0.248, *P* ≥ 0.173, see “Results”). AU arbitrary units (measured by changes in probe signal strength as animals move nearer or farther from the receiver [48]). ***P < 0.001

Acetazolamide is safe when given after ICH. Core temperature and blood pressure were stable after repeated doses (Fig. 1a, b). Acetazolamide did not affect hematoma size (Fig. 1; *P* = 0.858) or spontaneous home cage movement (Fig. 2a, *P* = 0.506). Physiological parameters monitored after a single dose in anesthetized animals showed no effect on blood pressure, heart rate, or oxygen saturation (Table 1). Blood glucose increased over time, as is known to occur during isoflurane anesthesia [31]. Finally, PO_2_ and PCO_2_ were 140.6 ± 9.9 and 52.3 ± 1.9 mmHg, respectively, when measured 1 h after the final dose of acetazolamide in animals used to monitor core temperature.

**Table 1:** Acute physiological parameters after 50 mg/kg acetazolamide in isoflurane-anesthetized rats (*n* = 4)

		Time post-injection	
Parameter	Baseline	0–30 min	30–60 min
Blood pressure (mmHg)	81.5 ± 1.1	77.9 ± 1.0	77.5 ± 2.8
Oxygen saturation (%)	97.0 ± 2.0	96.6 ± 1.9	98.1 ± 0.4
Heart rate (beats per minute)	328.3 ± 7.7	328.6 ± 6.6	327.8 ± 6.2
Blood glucose (mmol/L)	12.3 ± 1.4	18.4 ± 0.5***	20.5 ± 1.4**

**P < 0.01, ***P < 0.001 compared to baseline

ICH caused significant edema in the injured hemisphere (*P* < 0.001), which acetazolamide did not affect (Fig. 2b; *P* = 0.600). Consistent with our previous work [7, 14], there was no linear relationship between injured hemisphere water content and peak 1- or 30-min ICP or mean ICP in ICH rats (Fig. 2c, *r* = − 0.347 to − 0.248, *P* ≥ 0.173).

**Fig. 3:**
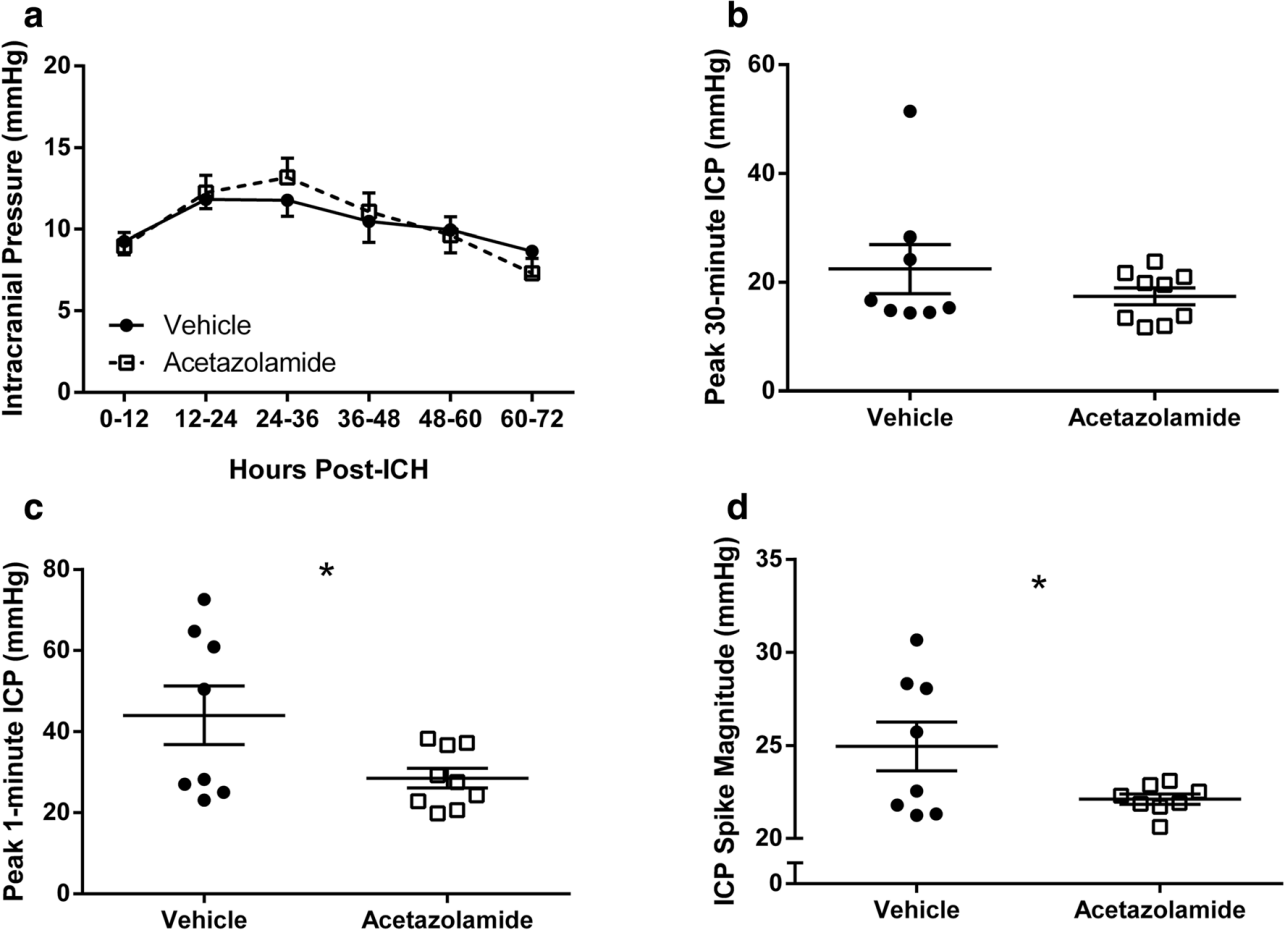
Effects of acetazolamide on ICP after ICH. **a** Acetazolamide did not affect mean ICP (shown as mean over 12-h epochs). Acetazolamide did not change peak 30-min ICP (**b**), but significantly reduced peak 1-min ICP (**c)** and the mean ICP of spikes above 20 mmHg (**d**). Mean ICP in uninjured rats using this technique is ~ 4–5 mmHg [13–15]. *P < 0.05

ICH increased ICP throughout the recording period as compared to uninjured rats where ICP is ~ 4–5 mmHg when measured with this technique [13–16]. ICP declined towards normal levels near the end of the recording period, consistent with our past work [7, 13]. Acetazolamide did not affect mean ICP (Fig. 3a; *P* = 0.952, group main effect) or 30-min peak ICP (Fig. 3b; *P* = 0.162). However, acetazolamide reduced transient ICP spikes; the 1-min peak ICP (Fig. 3c; *P* = 0.037) and the average magnitude of spikes above 20 mmHg (Fig. 3d; *P* = 0.041) were significantly less in drug-treated animals. One acetazolamide-treated animal did not have any spikes above 20 mmHg and was omitted from the latter comparison. Importantly, high ICP suppressed spontaneous activity; mean activity was significantly less when ICP was 20–40 mmHg (1.93 ± 0.16 AU; arbitrary units) than when ICP was 0–20 mmHg (2.31 ± 0.06 AU; *P* = 0.035). Note that for this analysis, “zero” activity values were excluded to avoid times of sleep.

**Fig. 4:**
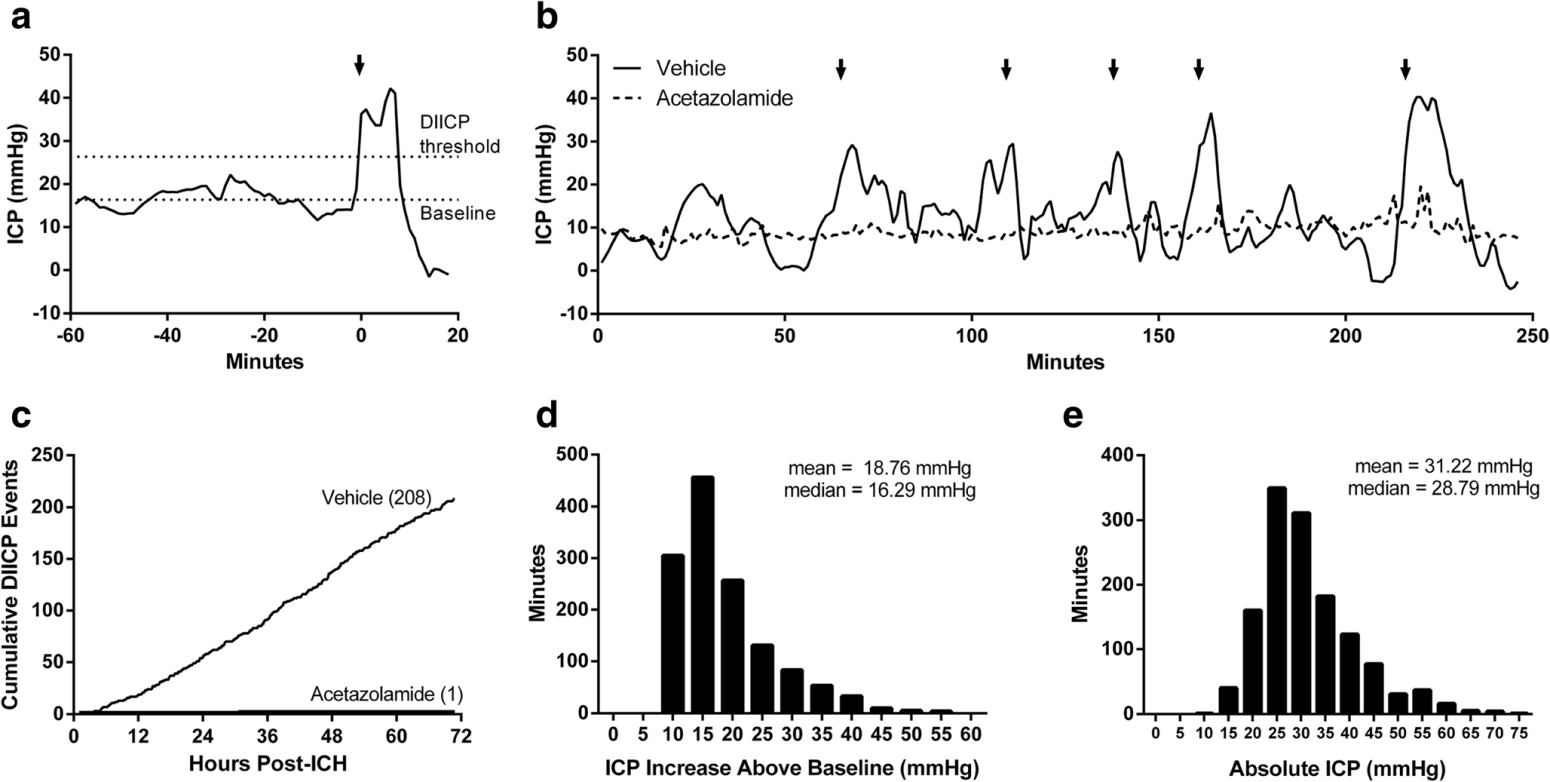
Disproportionate increases in ICP (DIICPs) after ICH. **a** An example of a DIICP in a vehicle-treated animal. Arrow indicates start of DIICP. Note the rapid increase and overcompensatory drop at the resolution of the event, which are indicative of poor intracranial compliance. **b** An example of multiple DIICPs over several hours in a vehicle-treated animal (solid line). Each arrow indicates the start of a discrete event. A representative ICP trace from an acetazolamide-treated animal is shown with a dashed line. Note the relative stability compared to the vehicle-treated animal. Also note that spikes were much less frequent and of smaller magnitude in the vehicle-treated animal (none exceeded the threshold to be considered DIICPs). **c** Acetazolamide nearly abolished DIICPs (*P* < 0.001, see “Results”). Numbers in parentheses indicate the total number of DIICP events for each group. **d**, **e** Histograms of ICP during DIICPs show that ICP increases tended to be rather large. Distribution of ICP during DIICPs is presented as an increase relative to the 60-min moving average baseline (**d**) and absolute ICP (**e**). Note that increases above ~ 20–25 mmHg are potentially lethal in rats and that ICP in uninjured rats is about 4–5 mmHg [7, 13–15]

ICH caused DIICPs of varying duration and magnitude (Fig. 4a–e). Acetazolamide nearly eliminated DIICPs (vehicle: 208 events across 8 animals, acetazolamide: 1 event across 9 animals, Fig. 4c, *P* < 0.001, comparing slopes of cumulative events between groups; examples of DIICPs from vehicle-treated animals are shown in Fig. 4a, b, a representative trace from an acetazolamide-treated animal is also shown in Fig. 4b for comparison). ICP increased considerably during DIICPs (typically > 25 mmHg, Fig. 4d, e). DIICP events lasted from 3 (the defined minimum) to 23 min (mean 6.42, median 5 min, interquartile range 3.75 min).

**Fig. 5:**
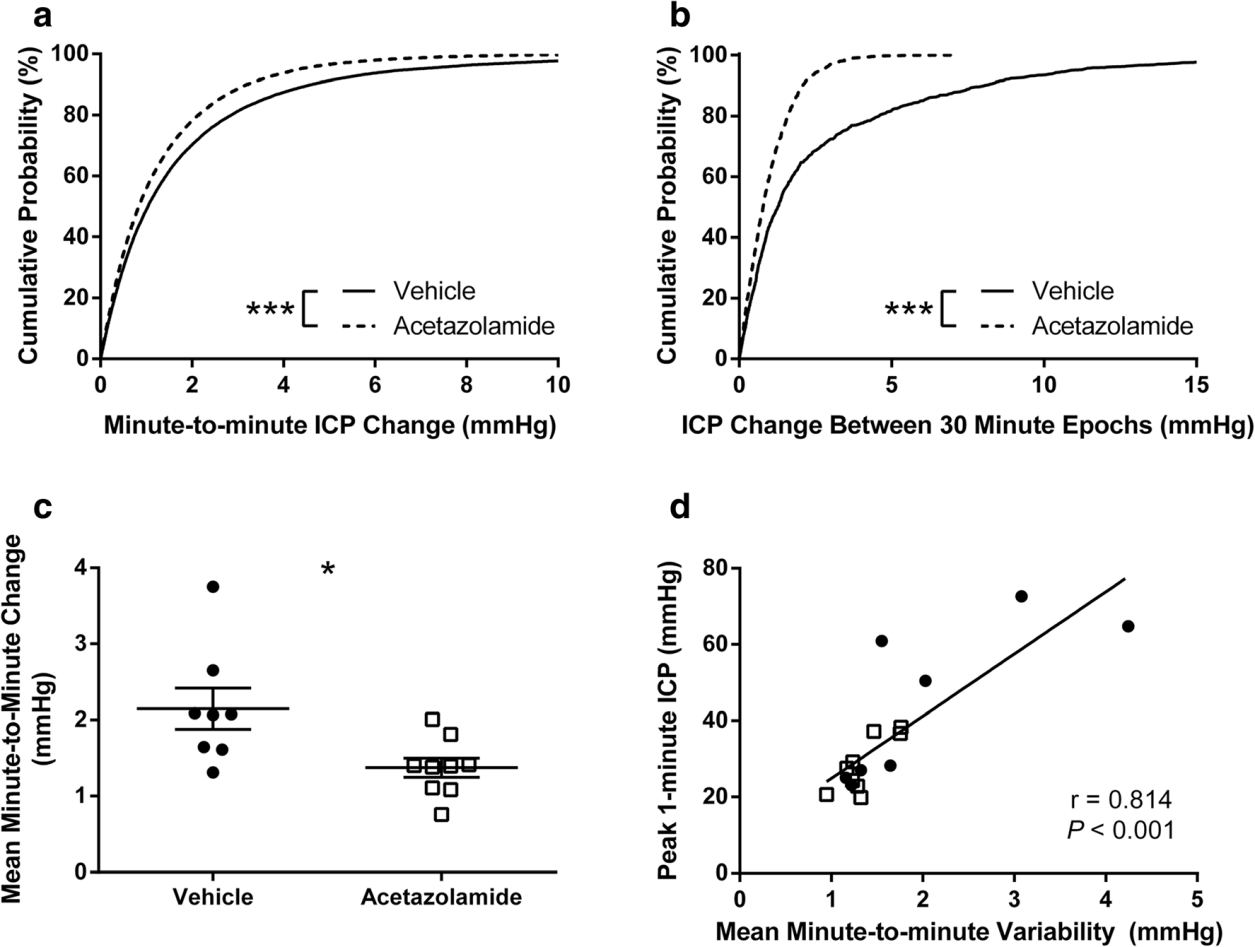
Acetazolamide reduced ICP variability. Cumulative probability plots of the magnitude of ICP change between minute-to-minute measurements (**a**) and 30-min epochs (**b**). Acetazolamide caused a significant leftward shift in curves at both timescales, indicating reduced variability and improved intracranial compliance. **c** Decreased mean minute-to-minute ICP change in acetazolamide-treated animals during the 30-min peak ICP of each animal further demonstrates that acetazolamide stabilizes ICP fluctuations in a high pressure state. **d** High ICP variability was associated with high peak and mean ICP (see “Results”). *P < 0.05; ***P < 0.001

Acetazolamide reduced ICP variability, demonstrating improved intracranial compliance, as indicated by marked leftward shifts in the cumulative probabilities of the magnitude of ICP change between 1- and 30-min epochs (Fig. 5a, b, *P* < 0.001). Importantly, acetazolamide decreased the mean minute-to-minute ICP variability during the 30-min peak ICP in each animal (Fig. 5c; *P* = 0.016). High ICP variability (poor compliance) was associated with high mean and peak ICP (1-min or 30-min variability vs. 1-min or 30-min peak ICP or mean ICP, *r* = 0.469 to 0.887, all *P* < 0.05, except 30-min variability vs. mean ICP where *P* = 0.058; Fig. 5d).

**Fig. 6:**
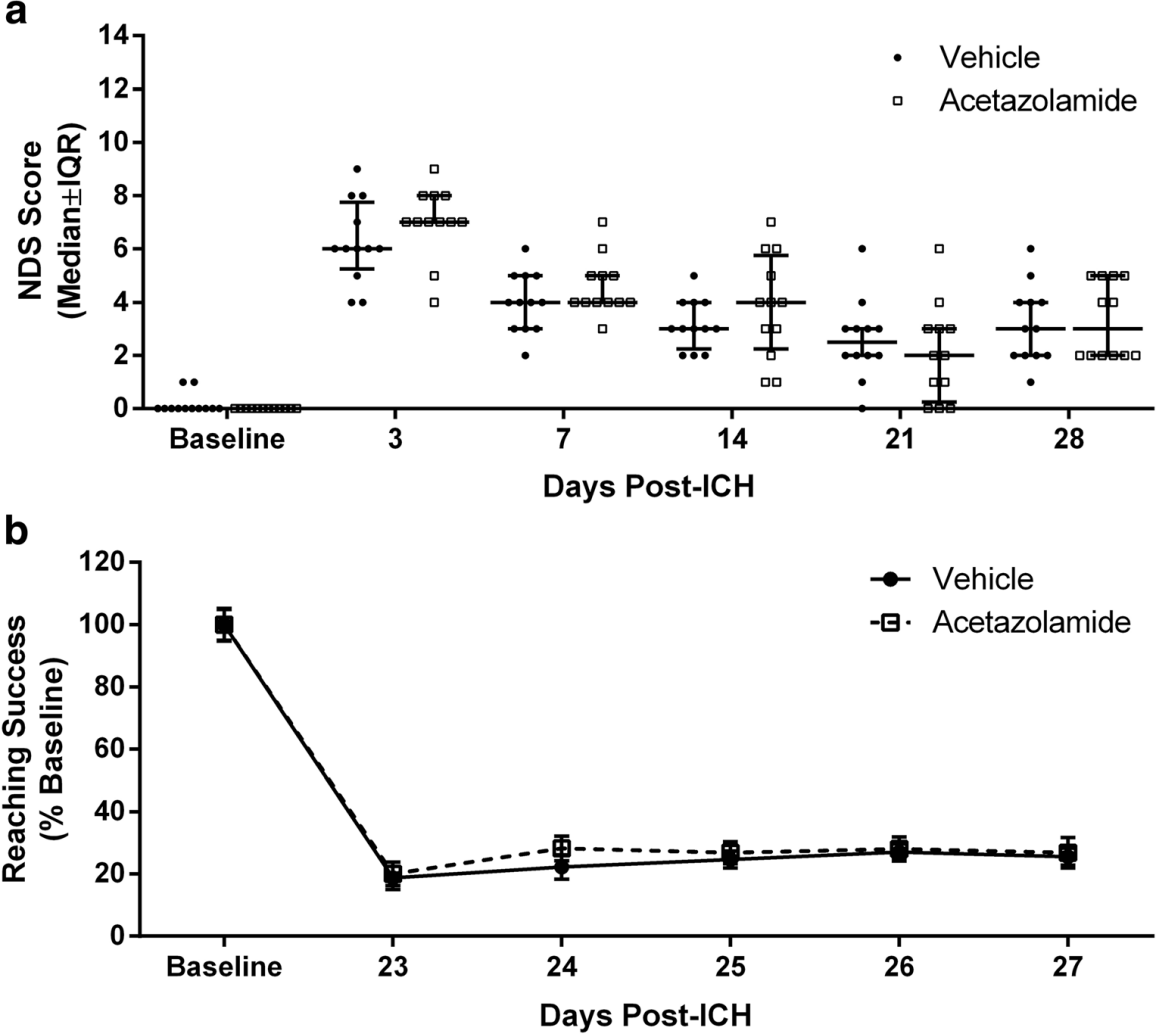
Acetazolamide did not improve behavioral outcome after ICH. **a** Repeated Neurologic Deficit Scale testing showed significant impairments after ICH, but no effect of acetazolamide. Possible composite scores range from 0 (no deficits) to 14 (greatest deficits). **b** Staircase reaching task data show significant impairments in reaching performance that were unaffected by drug treatment

**Fig. 7:**
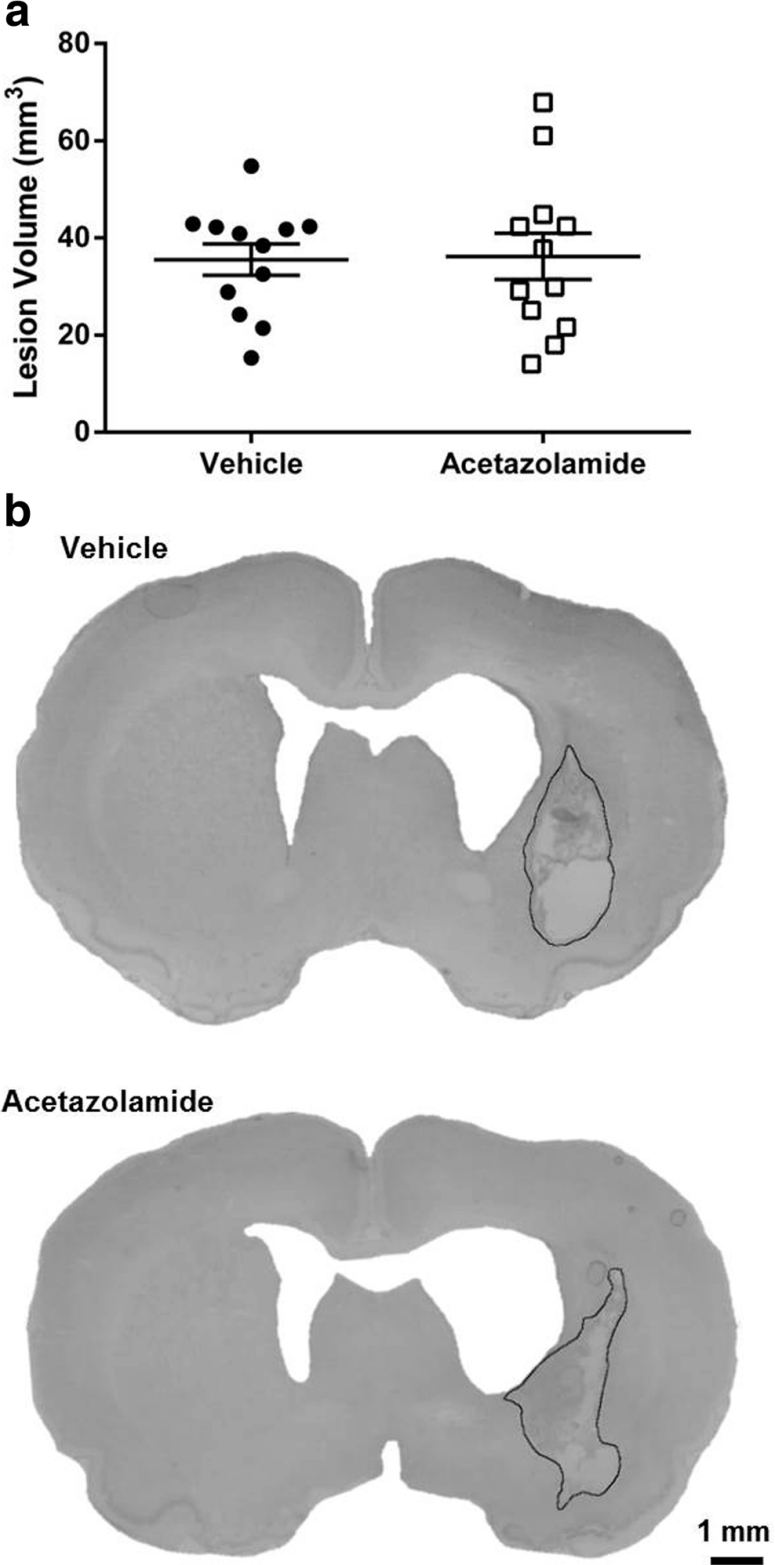
Acetazolamide did not affect lesion volume measured 28 days after ICH (**a**). **b** Representative grayscale coronal sections from the middle of the lesion stained with cresyl violet. Lesions are outlined in black. ICH caused tissue necrosis, cavity formation, and ventriculomegaly—all of which were accounted for in lesion volume measurements. Injury was restricted to the ipsilateral hemisphere. There was no damage to overlying cortex except near the path of the infusion needle

ICH caused significant impairments on the NDS and staircase tasks for the duration of testing (Fig. 6, *P* < 0.001, baseline vs. each post-ICH day for both tasks). Acetazolamide did not affect behavioral function measured with the NDS (Fig. 6a, *P* ≥ 0.178) or staircase tasks (Fig. 6b, *P* = 0.653, group main effect). As well, acetazolamide did not affect lesion volume (Fig. 7, *P* = 0.906). Linear regressions assessing lesion volume vs. NDS rank among all animals (rank ordered from least to most impaired over all test days, *r* = 0.355 and *P* = 0.089) and lesion volume vs. reaching success rank (averaged over all test days, *r* = 0.111 and *P* = 0.631) revealed no significant relationships. Similarly, there was no significant relationship between mean deficit rank (average rank between tasks) and lesion volume (*r* = 0.171, *P* = 0.458). Overall, lesion volume alone was a poor predictor of outcome.

## Discussion

Acetazolamide decreases CSF production and ICP in healthy animals and humans, and can provide benefit for patients suffering from idiopathic intracranial hypertension [32, 33]. Here, we report that acetazolamide ameliorates brief ICP spikes and restores intracranial compliance after experimental ICH but does not affect behavioral outcome or lesion size. Our results are not attributable to effects on intraparenchymal water content. Importantly, acetazolamide given 3 h after ICH does not worsen bleeding. Carbonic anhydrase inhibitors may provide a non-invasive means of reducing harmful ICP spikes without targeting edema, but whether such a treatment on its own would be beneficial is unclear.

Sudden large-magnitude ICP rises, as during DIICP events and other brief spikes, can reduce cerebral blood flow, cause herniation, and reflect a state of impaired intracranial compliance [5, 34–36]. The attenuation of ICP spikes and reduction of ICP variability by acetazolamide suggests that it restored intracranial compliance after ICH. This is perhaps most clearly demonstrated by the lower ICP variability in acetazolamide-treated animals during each animal’s 30-min peak ICP—a time when intact compliance mechanisms are critical for limiting damage potentially caused by high ICP. Indeed, the intracranial pressure-volume relationship is non-linear, and pulsatile pressure increases are much greater at higher ICP [35].

The transient ICP spikes we observed were quite large, typically exceeding 25 mmHg (Fig. 4e). Notably, current guidelines suggest that ICP be maintained below 20 mmHg in ICH and traumatic brain injury patients [5], and we have observed that sustained ICP increases above 20–25 mmHg in rats can cause death [13, 15]. As well, it is conceivable that large spikes could meaningfully reduce cerebral blood flow or otherwise impair brain function without overt trauma (herniation) [36]. Indeed, spontaneous movement declined during times of high ICP, suggesting some impact on behavioral function. However, we note that our previous study showed that, at least when averaged over 24 h epochs, collagenase-induced ICH only modestly lowered cerebral perfusion pressure [13]. Simultaneous monitoring of cerebral perfusion pressure and ICP at high temporal resolution would be ideal. Overall, it seems reasonable that the repeated ICP spikes we characterized in this study, although brief, could be harmful. Therefore, it is promising that acetazolamide nearly eliminated DIICPs and reduced the average magnitude of other large spikes. However, we found that mean ICP was not different between our vehicle and drug groups, despite large differences in short-timescale spikes. This discrepancy is likely due to the offsetting ICP declines that typically followed spikes, as is characteristic of poorly compliant systems (Fig. 4). It appears that relatively fast ICP changes are masked when averaged over long epochs. Many preclinical studies of ICP report mean ICP as their principle measure, although there is great interest in applying better predictive analyses in the clinical population [30, 37].

Brain hemorrhages and other ectopic cerebral masses can disturb CSF flow dynamics [38, 39]. Our results show that acetazolamide improved intracranial compliance and mitigated ICP spikes, but, likely due to the overcompensatory ICP drops that typically followed spikes, mean ICP averaged over long timescales was not different between treatment groups. This suggests that perturbed CSF dynamics after ICH might contribute to high ICP variability and large magnitude spikes. We speculate that a consequence of reducing CSF production with acetazolamide was to limit the disturbance in CSF flow, thereby dampening ICP fluctuations and ameliorating ICP spikes. Other means of restoring normal CSF dynamics (e.g., shunt) should similarly reduce ICP spikes.

Despite significantly reducing ICP spikes, acetazolamide did not improve behavioral outcome after ICH. There are several conclusions that could be drawn from this. First, it is possible that ICP does not influence outcome in this ICH model. This seems unlikely due to the considerable clinical evidence linking various measures of ICP to outcome [1–5] and observations within the collagenase model that high ICP can be lethal [13]. Second, brief ICP spikes may not be as predictive of outcome as high mean ICP. Perhaps, brief spikes are primarily dangerous when they cause herniation or subarachnoid hemorrhage, which we did not observe. Third, our behavioral tests may not have been able to detect any actual functional differences due to the somewhat large hemorrhages we induced. Indeed, impairments were rather severe and there was relatively little recovery of function; reaching success was < 30% of baseline 27 days after ICH, and there was little indication of improvement on the NDS task from day 7 to 28. However, the staircase and NDS tasks are among the most sensitive and widely used tasks for probing deficits caused by striatal hemorrhages [19]. Alternatively, any beneficial effects of acetazolamide might be more evident after more severe hemorrhages in which larger, more sustained ICP spikes occur. Indeed, the clinical population most likely to experience benefit from mitigating ICP spikes is that with severely raised ICP with high mortality, which we did not see in our model.

Consistent with previous work [7, 14], our data show that edema is not predictive of conventional measures of ICP such as mean and peak ICP, at least for a given injury severity. There does seem to be some correlation between edema and ICP over a larger range of injuries (i.e., across studies) and when data include sham-operated animals. Since water contained within the hematoma can contribute to brain water content measurements [14], such measurements are ideally done on only peri-hematoma tissue samples that exclude the hematoma. However, due to the irregular boundaries of the hematomas produced by collagenase infusion, this cannot be done with great accuracy in the model we used. Nonetheless, collagenase-induced ICH causes widespread edema relative to hematoma size [26]; we expect that hematoma-constrained water was a relatively small factor in our measurements.

Also of interest is whether hematoma size predicts ICP. Our past study using the whole blood infusion model found only a small and transient difference in ICP between varying hematoma volumes [14]. For example, increasing the blood infusion volume from 100 to 160 μL only further increased ICP by ~ 3–4 mmHg for about 12 h [14]. Relative to hematoma size, collagenase-induced ICH causes much larger and longer lasting ICP rises than blood infusion [7, 13, 14]. As discussed, these differences in the magnitude and pattern of ICP elevations are not completely explained by model differences in edema [7, 14]. At least in rodent models, it seems that edema and hematoma size alone are not precise predictors of ICP. Here, we found that compliance measures were correlated with mean and peak ICP, as well as the number of DIICP events (Fig. 6d, also see “Results”). Notably, mean 1-min ICP change, which reflects short timescale compensatory ability of the intracranial space, tended to be most strongly correlated with ICP. Compliance appears to predict future ICP better than other factors such as hematoma size and edema.

The ability of acetazolamide to reduce CSF formation and ICP is well-documented across species [11, 12, 21, 22, 27]. Recently, a single, very large dose of acetazolamide (~ 800 mg/kg, ~ two to three times the scaled maximum daily dose recommended in humans [24, 33]) was shown to reduce ICP by as much as 85% for about 5 h in uninjured anesthetized rats [40]. Such a large dose would likely cause significant metabolic acidosis and would therefore not be clinically valuable, especially with the need for repeated doses to target persistently raised ICP as in the present study [41]. As such, we did not assess those doses. Other work indicates that reduced CSF volume can last at least 24 h after a single 30 mg/kg dose in rats [27], and most studies have used 30–50 mg/kg (equivalent to a typical 500 mg dose in humans [24, 25]) with similar effects on ICP to that reported by the study using 800 mg/kg [11, 12]. The primary mechanism for the effects of acetazolamide is thought to be carbonic anhydrase inhibition, which interferes with bicarbonate transport in choroid plexus and thereby diminishes CSF production [11]. Other effects of acetazolamide in the CSF production pathway have been suggested, including actions on Na/K ATPase and aquaporins [40, 42].

Aside from affecting CSF production, acetazolamide is a vasodilator that can transiently increase cerebral blood flow [17]. While it is plausible that increased cerebral blood volume could acutely increase ICP, this does not appear to be the case. This is perhaps most clearly demonstrated in a study by Uldall and colleagues where ICP in anesthetized rats was continuously monitored before and after acetazolamide administration [40]. They observed a steady decline in ICP within 10 min of drug delivery with no indication of an ICP increase. However, another study described brief, modest ICP rises immediately after acetazolamide administration in a small number of animals, although ICP quickly declined [43]. Of course, it is possible that increased total cerebral blood volume due to vasodilation blunts the effects of CSF depletion on ICP, but the net effect is a reliably decreased ICP. Our data indicate that there was no difference in blood volume in the contralateral hemisphere or cerebellum 24 h after acetazolamide compared to vehicle administration, demonstrating that there is no lasting change in cerebral blood volume.

Potential deleterious side effects of reducing CSF production in order to treat high ICP should also be considered. For instance, CSF flow is implicated in waste removal from the brain [44]. Whether CSF flow has a role in removal of harmful hematoma breakdown components after ICH is unknown, but slowing such a process could be detrimental. As well, CSF acts as a compensatory reserve to maintain normal ICP. Significant reduction of CSF volume could result in below normal ICP, although low ICP is typically less harmful than high ICP [10]. Moreover, acetazolamide only decreases CSF production by about half at most [23], meaning that CSF production could be titrated by drug dose without concern of complete inhibition. Finally, acetazolamide and other carbonic anhydrase inhibitors can cause metabolic acidosis [41], which could be especially harmful with very large or chronic doses.

Previous work showed that acetazolamide may be neuroprotective after ICH [45]. Since that study found a neuroprotective effect after striatal infusion of 100 μL of blood, it is unlikely that it is due to an effect on ICP because the blood infusion model does not dangerously raise ICP, even with larger infusion volumes [14]. However, we note that in that study, acetazolamide was administered as an intracerebral co-injection with blood and sample size was only three per group for measures of cell death. In the present study, we gave acetazolamide systemically at a clinically relevant 3-h delay and did not observe any neuroprotective effect in a relatively large sample (12 per group). It is difficult to conclude whether carbonic anhydrase inhibition presents a useful and effective method for neuroprotection after ICH due to model, timing, dose, and administrative route differences between studies. As well, we note that while data from an in vitro astrocyte trauma model suggested that acetazolamide can attenuate cellular edema [46], our in vivo data found no effects of acetazolamide on brain water content after ICH (Fig. 2b) or in healthy rats (not shown).

Our study has some limitations. First, we did not directly assess CSF production. Nonetheless, acetazolamide is well-known to reduce CSF production across species, and its effectiveness in rats using the same or similar dose given systemically as in our study has been characterized [11, 12, 21, 27]. Moreover, measuring CSF production is technically difficult and not compatible with ICP monitoring [21]. As well, the marked reductions in ICP spikes and variability we observed indicate that acetazolamide did influence ICP with the dose, timing, and administration route we used. Second, we were unable to measure ICP and behavioral outcome in the same animals because our method for measuring ICP is most accurate during short-term (several days) experiments [15]. Long-term ICP monitoring has been achieved, but only with intermittent measurements that require the animal to be repeatedly anesthetized [47]. Such a method would not detect brief ICP spikes or permit assessment of ICP variability. A method for accurate, long-term, continual ICP monitoring that does not interfere with natural behavior is needed. Finally, in future studies, it would be ideal to combine ICP monitoring with simultaneous measurement of other parameters, such as tissue oxygenation and cerebral blood flow, but this is not currently feasible in freely moving rodents.

In summary, we show that acetazolamide mitigates brief ICP spikes and improves intracranial compliance without affecting hematoma formation, edema, mean ICP, behavioral outcome, or lesion size after collagenase-induced ICH in rats. We speculate that impaired CSF flow dynamics after ICH may contribute to ICP spikes and that acetazolamide ameliorates ICP spikes by diminishing CSF production. Overall, these findings might suggest that brief ICP spikes and intracranial compliance are poor predictors of behavioral outcome in the collagenase model, but additional work is needed to clarify precise links between ICP measures and outcome across a broader range of situations. Despite the lack of behavioral benefit in our study with moderate hematoma size, reducing harmful ICP spikes may reduce mortality in patients with large hemorrhages. Combining acetazolamide with existing treatments that target edema may more potently protect against ICP spikes.
